# *γδ* T Cell and Other Immune Cells Crosstalk in Cellular Immunity

**DOI:** 10.1155/2014/960252

**Published:** 2014-03-06

**Authors:** Ying He, Kangni Wu, Yongxian Hu, Lixia Sheng, Ruxiu Tie, Binsheng Wang, He Huang

**Affiliations:** Bone Marrow Transplantation Center, The First Affiliated Hospital, Zhejiang University School of Medicine, 79 Qingchun Road, Hangzhou 310003, China

## Abstract

*γδ* T cells have been recognized as effectors with immunomodulatory functions in cellular immunity. These abilities enable them to interact with other immune cells, thus having the potential for treatment of various immune-mediated diseases with adoptive cell therapy. So far, the interactions between *γδ* T cell and other immune cells have not been well defined. Here we will discuss the interactivities among them and the perspective on *γδ* T cells for their use in immunotherapy could be imagined. The understanding of the crosstalk among the immune cells in immunopathology might be beneficial for the clinical application of *γδ* T cell.

## 1. Introduction

*γδ* T cell accounts for a small group, which is less than 10% of the T cell pool in healthy human individuals [1]. Strong evidence demonstrates that *γδ* T cell participates as part of both innate and adaptive immunity. On activation, these cells can expand markedly and display various effector functions in immune responses. For example, chemokines and inflammatory cytokines release, potent cytolytic activity against tumor or microbial pathogens, and immunologic memory generation. These characteristics may contribute to the cell-cell contact manner of *γδ* T cell with other immune cells.

Empirical studies demonstrate that *γδ* T cells recognize transformed cells, microbial or tumor-expressed antigens, and then develop the immune surveillance functions [2]. It is clear that *γδ* T cells are able to respond to pathogen-associated molecular patterns of infection and autoimmunity. Virtually, their functions are not limited to antitumor or antiviral actions but also involved in modulating immune system homeostasis [3]. And this homeostasis may depend on the cross-reactivities between *γδ* T cells and their neighbour immune cells [4]. Selective stimulation of *γδ* T cells* in vivo* for antitumor therapy was accompanied by unexpected expansion of natural killer cells (NK cells) in a clinical trial [5]. It cannot be clearly distinguished whether the antitumor effect is produced by anyone of these two cells or there exists a synergy effect between them. The cell-cell interactions between *γδ* T cell and other immune cells are largely unknown and therefore, it is hard to assess their roles for the example above.

In recent clinical studies, suppressive regulatory T cells (Tregs) have been infused into patients to control the activation of alloreactive T lymphocytes after allogeneic haematopoietic stem cell transplantation (AHSCT) [6, 7]. Adoptive transfer of different immune cell subsets for treating cancer and/or immune-mediated diseases is increasingly being tested in clinical trials. The challenge for this therapy is how to efficiently exert regulatory effects on the target cells. As described above, *γδ* T cell plays an important role in immune response and thus has the potential for such immune-based therapies. Therefore this raises the question how the *γδ* T cell communicates with other immune cells. Understanding their crosstalk may be beneficial for the development of immunotherapeutic strategies.

## 2. *γδ* T Cell and *αβ* T Cell

T lymphocytes express either *αβ* or *γδ* T cell receptor heterodimers. Previous works have revealed the similarities between *γδ* T cell and the more populous *αβ* T cell in some aspects, such as cytolysis [8] and secretion of multiple cytokines [9]. These properties of *γδ* T cells permit them to regulate many types of immune response and cellular activities, including those of the predominant subsets-*αβ* T cells. A variety of studies show that V*δ*2^+^ T cells act like professional antigen-presenting cells (APCs) to take up and present antigens (Ags) to human *αβ* T cells [10, 11], as well as in some mouse *γδ* T cells [12]. This capacity for Ag presentation by *γδ* T cells is considered to be a cooperative way in immune defense. Furthermore, the isopentenyl pyrophosphate- (IPP-) activated V*δ*2^+^ T cells can promote proliferation and differentiation of naïve CD8^+^ *αβ* T cells [8] and even enhance the interferon (IFN)-*γ* production from autologous colonic *αβ* T cells [13]. However, all of these results are derived from* in vitro* experiments. Still, little is known about whether these cell-cell interactivities can be investigated under both* ex vivo* and* in vivo* conditions.

From a mouse model, *γδ* T cell depletion by anti-*γδ* T cell receptor (TCR) monoclonal antibody GL3 followed by concomitant elevated numbers of *αβ* T cells was described [14]. Likewise, the CD8^+^ T cell-mediated liver damage in Listeria-infected TCR*δ* ^*−/−*^ mice could be prevented by transferred with *γδ* T cells, and this effect may depend upon the ability of *γδ* T cells to reduce tumour necrosis factor (TNF)-*α* secretion or expansion of CD8^+^ T cells [15]. Obviously, there is homeostatic competition between *αβ* T cells and *γδ* T cells* in vivo*, and the IL-15 production and* trans*-presentation by dendritic cells (DCs) may be one possible mechanism for this activity [16]. Nevertheless, we cannot conclude that *γδ* T cells only have immunosuppressive effects on *αβ* T cells* in vivo*. An interesting result shows that inactivation and/or depletion of V*γ*4^+^ T cells in the complete Freund's adjuvant- (CFA-) treated mice lead(s) to significant decreased number of *αβ* T cells as well as reduced TNF-*α* and IFN-*γ* production [17]. These results provide the concept that the modulation effects of *γδ* T cell on *αβ* T lymphocyte are mysterious. There has been no explanation so far for such discrepancy.

By studying the *αβ* lymphocytes, it has been found that CD8^+^ *αβ* T cells potently inhibit *γδ* T cells expansion and compete for essential cytokine stores when both of them are cotransferred into TCR*β* ^*−/−*^/*δ* ^*−/−*^ mice [4]. Similar results are seen in the CD4^+^CD25^+^ regulatory *αβ* T cells, a subset of *αβ* T cells, and they also have the capacity to suppress the expansion and functions of *γδ* T cells [18]. However, when adoptive *αβ* T cells (or CD4 T cells) were transferred into TCR*β* ^*−/−*^ mice, these cells positively restored interleukin-17^+^ (IL-17^+^) *γδ* T cells generation, thereby implicating the supportive role of CD4^+^ T cells for IL-17^+^ *γδ* T cells [19]. Taken together, these data demonstrate that the reciprocal effects between *γδ* T cells and *αβ* T cells are debatable. The possible reasons for the contradictory data may be the different functions of subsets of *γδ* T cells, and the use of heterogeneous or homologous T lymphocytes also induces various immune responses.

## 3. *γδ* T Cell and B Cell

In the *γδ* T cell/B cell coculture experiments, the amounts of some immunoglobulins production increased remarkably [20, 21]. It is reported that *γδ* T cells also can collaborate with B cells to support the germinal center formation [22, 23]. In addition, an* in vivo* finding showed that TCR*α* ^*−/−*^ mice still efficiently developed normal germinal center and produced immunoglobulins (Igs), which thus prompts the hypothesis that *γδ* T cells might provide help for B cells [24]. In fact, not only the activation of immune responses but also the promotion of B cell maturation by *γδ* T cell can be investigated in human [25]. It is clear that *γδ* T cell is responsible, at least in part, for support of B cell functions. And recent investigations suggest that production of great amounts of cytokines from *γδ* T cells may influence B cell responses in humoral immunity [26, 27].

Although most studies state the promotion of B cell activities by *γδ* T cell, the limitation has been found under some circumstances. When mice received stimulation with ovalbumin (OVA) repeatedly, their splenic *γδ* T cells can inhibit the primary IgE production [28]. These regulatory functions are speculated to be mediated by different *γδ* T cells. For example, innate V*γ*1^+^ T cells, including V*γ*1V*δ*5^+^ subsets, can enhance IgE responses, whereas acquired V*γ*4^+^ T cells repress the *αβ* T cell-dependent antigen-specific IgE responses [29]. Collectively, these findings suggest that *γδ* T cells have complex immunomodulatory functions upon B lymphocytes similar to that on *αβ* T cells and this phenomenon may be related to the subsets of *γδ* T cells.

To understand whether the B cell can affect *γδ* T cell, multiple studies are attempting to disclose their interactions. It is reported that allogeneic Epstein-Barr virus- (EBV-) transformed B cell lines augment the proliferation of V*δ*1^+^ T cells [30]. Further insights into the mechanism indicate that the isolated human peripheral blood B lymphocytes can induce proliferative response of V*δ*1^+^ T cells, and it may be attributed to the expression of B7 and CD39 molecules on the surface of activated B cells [31]. Because *γδ* T cells undergo immune response independent of major histocompatibility complex (MHC) molecules, mutant EBV-transformed B cell lines lacking MHC molecules still can present the bacterial phosphoantigens (PAgs) to *γδ* T cells for other T cells activation [32, 33]. Accordingly, rational combination of these two cell types for immunotherapies would be considerable.

## 4. *γδ* T Cell and NK Cell

Similar to another lymphoid cell-NK cell, *γδ* T cell exerts immune functions in the antibody-independent and non-MHC-dependent manners. Although the NK-cell-like functions of *γδ* T cell are well characterized, the interactivity between *γδ* T cell and NK cell remains enigmatic. Chapoval et al. showed that *γδ* T cells were indispensable for regulation of NK-cell antitumor responses [34]. Afterwards they further identified that, in the TCR-*δ* ^*−/−*^ mutant mice, early IFN-*γ* production seriously decreases in the listeriosis-infected group, and they also confirmed that NK cells were the critical producer of IFN-*γ* [35]. Maniar et al. have described a similar result that zoledronic acid-activated *γδ* T cells can lead to enhancement of NK cell-mediated tumor cytotoxicity [36]. Recently, the DC-like cell-dependent NK cell cytokine production has been found to be controlled by *γδ* T cells [37]. Thus these results manifest that lack of *γδ* T cells may lead to impaired NK cells activation.

In turn, when stimulated by the antigens from* M. tuberculosis*, activated NK cells can improve *γδ* T cells proliferation [38]. However, under* in vivo* condition, NK cells play a suppressive role in regulating *γδ* T cells expansion in the absence of *αβ* T cells [4]. Such phenomenon demonstrates that, different from the* in vitro* condition, the interplay between NK cells and *γδ* T cells may lie on the competitive advantages of the cells under* in vivo* condition. Actually, NK cells or *γδ* T cells use different mechanisms to mediate cytotoxicity functions, so the combination of them for immunotherapy would be considered. Understanding of their correlation may provide the concept for how to enhance the antitumor functions by immune cells.

## 5. *γδ* T Cell and Monocyte/Macrophage

Monocytes play a role in immune defense and after travelling to tissues, they mature and differentiate into macrophage populations. At the sites of infection/inflammation, circulating *γδ* T cells and monocytes are rapidly recruited to eliminate infected or transformed cells. Several investigations demonstrate that they do not function alone but affect each other. It is previously shown that microbe-responsive V*γ*9V*δ*2 T cells can induce monocytes differentiating into inflammatory DCs, which further results in production of inflammatory mediators and antigen-presenting functions of these differentiated monocytes [39]. In turn, some evidence suggests that* M. tuberculosis*-infected monocytes are potent in inducing *γδ* T proliferation [40] and processing the* M. tuberculosis* for mycobacterial antigen-specific CD4^+^ *αβ* and *γδ* T cells [41]. After being incubated with* M. tuberculosis* Bacillus Calmette-Guérin (BCG), infected monocytes promote the cytotoxic activity of *γδ* lymphocytes [42]. Additionally, the N-BP drug zoledronic acid-treated monocytes can effectively trigger activation of *γδ* T cells [43]. But in atopic dermatitis (AD) patients, once contacted with activated monocytes, NK and *γδ* ^+^ T cells specifically undergo apoptosis associated with reduction of type 1 cytokine production [44]. It seems that different pathological states of monocytes may account for the fate of *γδ* T cells.

In the case of macrophage, when incubated with* Mycobacterium tuberculosis*-derived products, macrophages release chemokines to efficiently recruit *γδ* T cells as well as regulating the latter's function [45]. As for *γδ* T cell, different subsets may be responsible for their specific functions. In the mice infected with influenza A virus, V*γ*6V*δ*1^+^ T cells might contribute to the initial recruitment of macrophages whereas V*γ*4^+^ and V*γ*1^+^ T cells mediate elimination of macrophages [46]. For preventing the hyperinflammatory responses, *γδ* T cells exert cytotoxic activity against activated macrophages [47]. Similarly, human or avian influenza virus-infected macrophages were killed by PAg-expanded *γδ* T cells for virus clearance [48]. However, human peripheral V*γ*9V*δ*2 T cells have been shown to efficiently induce TNF-*α* and IL-1*β* production of BCG-infected macrophages [49]. Obviously, different subtypes of *γδ* T cells have their specific functions to regulate the activities of macrophages.

## 6. *γδ* T Cell and DC

Unlike mouse *γδ* T cells, human *γδ* T cells distribute in the peripheral blood, spleen, lymph nodes, or the intraepithelial lymphocytes in intestine [50], but rare in human skin [51]. Various subsets of *γδ* T cells scatter in a disparate anatomic location and assume distinct functions [52]. The *γδ*1^+^ T cell population abundant in lymphoid tissues markedly blocks the maturation and inhibits the function of DCs [53]. Interestingly, the V*δ*2^+^ T cells, which consist of most human peripheral blood *γδ* T cells, seem to react in another way for DCs. When cocultured with V*δ*2^+^ T cells isolated from human blood samples, the maturation of immature DCs (iDCs) is shown to be potentiated [54], and the consistent phenomenon has been found in mice [55]. Furthermore, *γ*9*δ*2TCR-transduced *αβ* T cells efficiently promote the maturation of DCs [56]. Apart from the induction of maturation of DCs, V*γ*9V*δ*2 T cells have been identified for their ability for restoring* Brucella*-infected DCs function [57]. It is also reported that *γδ* T cells enhance DCs activation for the production of IL-12 [58]. The possible mechanism of *γδ* T cell for the induction of DC activation may be due to recognition of the cell-surface molecules or inflammatory cytokines, such as CD1 [59], CD86 [60], CD40 [58], and IFN-*γ* [61]. However, a recent study found that the immunosenescence could be induced in the DCs by *γδ* Treg cells [62]. Therefore, it is plausible that diverse effects of *γδ* T cells on DCs may rely on different *γδ* subsets.

It is investigated that DCs induce the cell cytokine production of freshly isolated V*γ*9V*δ*2 T cells [54], and they have the potent capacity to expand peripheral blood V*δ*2^+^ T cells [63] or support the development of V*γ*4^+^ *γδ* T cells from the spleen [64]. Moreover, mycobacteria can induce V*δ*2 T cell antitumour responses indirectly via a specific subset of DCs [65]. These* in vitro* studies suggest that the modulation effect of DC on *γδ* T cell is definite no matter what type of the *γδ* T cell is. In tuberculosis patients,* M. tuberculosis*-infected DCs selectively induced expansion of phenotypically immature, central memory-type V*γ*9V*δ*2 T cells [66]. Even though, in the patients with multiple myeloma (MM), zoledronic acid-treated DCs are potent in activating autologous *γδ* T cells [67], the above-mentioned findings, not only* ex vivo* but also* in vivo* study, are all suggestive of the promotion effects of DCs on *γδ* T cells regardless of the latters' subtype.

## 7. *γδ* T Cell and Granulocyte

During inflammatory or infectious processes, granulocytes and *γδ* T cells promptly accumulate at the site of inflamed tissues and eradicate the bacteria, virus, or transformed cells. It is established that *γδ* T cells partially resemble the granulocytes in cytotoxic activities against pathogens. Understanding of their cross-reactivity in the immune response might pave the way for immunotherapies. Neutrophils constitute most of the granulocytes; hence the investigations on the activities of granulocytes are focused on this population. HMB-PP stimulated V*γ*9/V*δ*2 T cells have been reported to induce neutrophil survival and activation depending on the number of *γδ* T cells [68]. Similarly, IPP or zoledronic acid-stimulated peripheral blood V*γ*9V*δ*2 T cells were observed to induce phagocytosis and migration of neutrophils [69], as well as granules release from activated granulocytes [70]. In addition, limbal epithelial *γδ* T cells [71] and hepatic IL-17A^+^CD3^+^ *γδ* TCR^+^ cells [72] have the capacity to regulate early infiltration or accumulation of neutrophils.

However, discrepant result reported that, in a mouse model of sepsis, there was a reverse association of the cell number between *γδ* T cells and neutrophils, and *γδ* T cells rapidly killed lipopolysaccharide- (LPS-) treated neutrophils [73]. At the presence of LPS—the major component of the membrane of Gram-negative bacteria—hypertonic saline leads to the increased elimination of inflammatory neutrophils by *γδ* T cells [74]. These controversial results have not yet been well explained so far, and it seems to be attributed to the status of neutrophils. Unfortunately, the function of neutrophils on *γδ* T cells has rarely been reported and the interaction between them should be further clarified.

## 8. *γδ* T Cell and Mesenchymal Stromal Cell

Mesenchymal stem cells (MSCs) are a minor subset residing in several tissues, generally isolated from bone marrow. The ability of MSC to regulate other major immune cell populations has been demonstrated in numerous studies, but only a few studies elaborate the interplay between *γδ* T cells and MSCs. The immunomodulatory activities of MSCs on T cells have been further investigated since it was confirmed that MSCs could inhibit the T lymphocytes proliferation either in mixed lymphocyte reactions or under the stimulation of polyclonal activators [75]. As recently reported, human MSCs inhibit the proliferation and immune responses of V*γ*9V*δ*2 T cells through prostaglandin E2 (PGE2) [76]. In addition, MSCs can suppress the expansion of V*δ*2^+^ T cells without affecting the functions of cytotoxicity or antigen processing/presentation to CD4^+^ T cells by V*δ*2^+^ T cells [77, 78]. The inhibitory role of MSC on *γδ* T cell appears to be determined by the MSC-derived molecules [79]. It is interesting to note that when infection or organ injury develops, *γδ* T cells increase the recruitment of MSCs to the site of infection [80]. Immunosuppressive effect of MSC is supposed to contribute to restoring tissue homeostasis whereas *γδ* T cell exerts defending functions against pathogens and tumors. Therefore, the use of MSC or *γδ* T cell in adoptive immunotherapy should carefully be taken into consideration for their interaction.

## 9. Concluding Remarks

**Figure 1:**
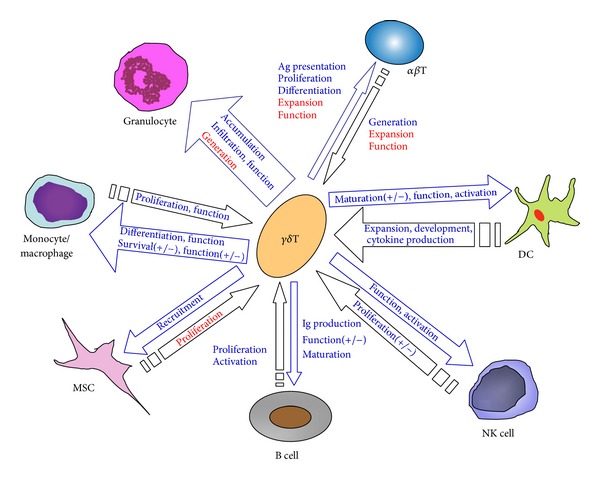
Interactions between *γδ* T cell and other immune cells. The positive effects are depicted in blue font, while the negative effects are in red. The mathematical symbol (+/−) indicates that the contradictory effects are observed between *γδ* T cell and other immune cells. MSC: mesenchymal stem cell; DC: dendritic cell; NK cell: natural killer cell; Ag: antigen; Ig: immunoglobulin.

A growing set of data has clearly identified that *γδ* T cells play different roles in immune responses, thus providing a promising candidate for treatment of many diseases. More recently, *γδ* T cells are making their way into clinical trials. In this review, the interactions between *γδ* T cell and other immune cells have been discussed (Figure 1), and there is the prospect that *γδ* T cells are potential for treating immune-mediated diseases. Unfortunately, current experimental data is almost derived from* ex vivo* studies or animal models, and there are extremely limited observations of the interaction between *γδ* T cells and other immune cells in human body. This would impede the application of *γδ* T cells for clinical therapeutics in the future. Therefore, more preclinical and clinical investigations of *γδ* T cells are needed for making effective strategies to harness immune responses in various diseases.

By studying the effects of *γδ* T cells on other immune cells, *γδ* T cells reveal a dual role so that it is not definite whether they would display active or suppressive function in immune activities. That *γδ* T cells are prone to be the positive or negative effectors may depend on their subtype or internal homeostasis. In this regard, strategies to regulate the interaction of these cells should be targeted on specific subsets as well as environmental factors. With regard to the modulation of other immune cells on *γδ* T cell, most of them have the role of supporting the function of *γδ* T cell except the inhibitive effects from MSC. Thus, the balance between *γδ* T cells and their neighbour immune cells should be considered sufficiently in the adoptive cell therapies for treating cancer or immune-related diseases.
